# Subspecies Distribution, Polymicrobial Associations, and Antimicrobial Resistance in Clinical Acinetobacter Isolates: A Prospective Cross-Sectional Study From a Tertiary Care Hospital in India

**DOI:** 10.7759/cureus.111693

**Published:** 2026-06-28

**Authors:** Jayaprada Rangineni, Udithi Bandaru, Visweswara R Guthi, Joshi S Jayapal Prabhudass, Supraja Indukuri

**Affiliations:** 1 Microbiology, Sri Venkateswara Institute of Medical Sciences, Tirupati, IND; 2 Microbiology, Sri Venkateswara Institute of Medical Sciences, Sri Padmavathi Medical College for Women, Tirupati, IND; 3 Community Medicine, Sri Venkateswara Institute of Medical Sciences, Sri Padmavathi Medical College for Women, Tirupati, IND

**Keywords:** acinetobacter, antimicrobial susceptibility, multidrug resistance, polymicrobial association, subspecies

## Abstract

Introduction

The global rise of multidrug-resistant *Acinetobacter* (*A.*), particularly *A. baumannii*, is well recognized. However, real-world data linking subspecies distribution with resistance patterns and polymicrobial dynamics in real-world tertiary care settings remain limited. This study addresses this gap by providing a comprehensive and clinically relevant profile of *Acinetobacter *isolates, emphasizing species-specific resistance patterns and polymicrobial associations.

Methods

A prospective cross-sectional study was conducted over six months (July-December 2023) in a tertiary care teaching hospital in South India. Clinical specimens yielding *Acinetobacter *species, including polymicrobial cultures, were analyzed. Antimicrobial susceptibility testing (AST) was performed using VITEK 2 COMPACT (BioMérieux, Marcy-l'Étoile, France) and Kirby-Bauer according to Clinical and Laboratory Standards Institute (CLSI) 2022 guidelines. The minimum inhibitory concentration (MIC) for polymyxin B was determined using microbroth dilution. Data were analyzed using Statistical Package for the Social Sciences (SPSS) Statistics version 21 (IBM Inc., Armonk, New York).

Results

Among 181 isolates, *A. baumannii* was the most prevalent, 118 (65.2%), followed by *A. lwoffii*, 39 (21.5%), and *A. haemolyticus*, 24 (13.3%). A strong predominance was observed in the intensive care unit (ICU), which accounted for 143 (79.0%) isolates. A key finding was the clear variation in resistance profiles across subspecies. *A. baumannii* demonstrated very high multidrug‑resistant (MDR), 102 (86.4%), and extensively drug‑resistant (XDR) 16 (13.6%) rates, whereas non‑*A. baumannii* species exhibited substantially lower resistance levels. Carbapenem resistance remained alarmingly high, with meropenem resistance at 145 (80.1%) and imipenem resistance at 124 (68.5%) across both ICU and non‑ICU settings, suggesting significant institutional selection pressure. Despite this, susceptibility to polymyxin B, 168 (92.8%), and tigecycline, 172 (95.0%), remained largely preserved. Polymicrobial infections were identified in 28 (15.5%) cases and showed a significant association with species type, with *A. lwoffii* exhibiting higher co‑isolation rates than *A. baumannii*. The most common co‑pathogens were *Pseudomonas* and *Klebsiella* species.

Conclusion

This study demonstrates clear species-level differences in both resistance and polymicrobial behavior within *Acinetobacter*, emphasizing the clinical importance of routine subspecies identification. The combination of high carbapenem resistance across care settings and species-specific polymicrobial trends highlights the need for precision antimicrobial stewardship, early targeted therapy, and unit-specific antibiograms. Together, these findings offer practical evidence to guide improvements in empiric treatment strategies in high-risk hospital settings.

## Introduction

*Acinetobacter* (*A.*) species (spp.), particularly *A. baumannii, *have become major nosocomial pathogens due to their ability to survive on hospital surfaces, colonize medical equipment, and accumulate multidrug resistance mechanisms across numerous antimicrobial classes [1]. Their impact is most evident in intensive care settings where mechanical ventilation, invasive procedures, and prior antibiotic exposure increase vulnerability and complicate empiric therapy [2]. In such high-risk environments, rapid species identification and strong local surveillance are essential to guide infection control practices and support early, appropriate treatment.

Rising resistance to cephalosporins, aminoglycosides, and carbapenems has narrowed therapeutic options, often restricting management to agents such as polymyxins and tigecycline [2,3]. This challenge is amplified in polymicrobial infections, common in device-associated respiratory and wound samples, where coexisting non-fermenters and *Enterobacter* may necessitate broader initial therapy until culture results allow de-escalation [3]. Understanding local subspecies distribution, patterns of polymicrobial involvement, and resistance profiles is therefore critical for optimizing empiric strategies in tertiary care settings.

*Acinetobacter* often coexists with other hospital-acquired pathogens in device-rich areas, and its strong environmental persistence contributes to mixed organism colonization [4,5]. Such polymicrobial patterns add complexity to clinical decision-making and highlight the need for contextual microbiological data.

This prospective cross-sectional study, conducted from July to December 2023 in a tertiary care teaching hospital in South India, aimed to characterize subspecies distribution of *Acinetobacter *isolates, quantify polymicrobial infections, analyze antimicrobial susceptibility with emphasis on carbapenems and last-line agents, and compare patterns across patient demographics and hospital locations. The study provides locally relevant evidence to inform infection prevention strategies and antimicrobial stewardship in high-risk clinical settings.

## Materials and methods

Study design and setting

A prospective cross-sectional study was conducted in the department of microbiology at a tertiary care teaching hospital, Sri Venkateswara Institute of Medical Sciences (SVIMS) Hospital, in Tirupati, Andhra Pradesh, India, from July to December 2023. The study evaluated the distribution of *Acinetobacter* spp., polymicrobial infections involving *Acinetobacter*, and antimicrobial susceptibility patterns across demographic and clinical variables.

Ethical approval

This laboratory-based study used de-identified data from routine diagnostic specimens without additional interventions. No personal identifiers were accessed. The Institutional Ethics Committee approved the study (approval number: 1429/2023) and waived the requirement for informed consent.

Eligibility criteria

All clinical specimens yielding *Acinetobacter *spp., including polymicrobial cultures, were included. Exclusion criteria were non-*Acinetobacter *non-fermenting Gram-negative bacilli, contaminants or commensals, and polymicrobial cultures without Acinetobacter or dominated by another pathogen.

Specimen processing and identification

A single clinical specimen was collected from each patient, including blood, urine, pus or wound swabs, sputum, endotracheal (ET) aspirates, cerebrospinal fluid (CSF), and other body fluids. All specimens were processed using standard microbiological protocols. Samples were inoculated onto blood agar, nutrient agar, and MacConkey agar plates and incubated aerobically at 37 °C. Culture plates were examined after 24 and 48 hours for bacterial growth.

Non-lactose-fermenting isolates underwent biochemical testing, including Gram staining, motility, catalase and oxidase tests, triple sugar iron (TSI) test, oxidation-fermentation test, growth at 37 °C and 42 °C, arginine dihydrolase activity, hemolysis, and chloramphenicol sensitivity. Species identification was performed using the VITEK 2 COMPACT system (bioMérieux, Marcy-l'Étoile, France).

Antimicrobial susceptibility testing

Antimicrobial susceptibility testing (AST) was performed using the VITEK 2 COMPACT system and Kirby-Bauer disc diffusion on Mueller-Hinton agar, following Clinical and Laboratory Standards Institute (CLSI) 2022 guidelines [6]. Twelve antibiotics were tested: amikacin (30 µg), cefotaxime (30 µg), cefoperazone/sulbactam (75/10 µg), ciprofloxacin (5 µg), gentamicin (10 µg), cotrimoxazole (1.25/23.75 µg), piperacillin/tazobactam (100/10 µg), meropenem (10 µg), ceftazidime (30 µg), aztreonam (30 µg), imipenem (10 µg), and polymyxin B (300 units), using the Kirby-Bauer disk diffusion method on Mueller-Hinton agar.

Quality control strains included *Escherichia coli* (*E. coli*) ATCC 25922 and *Pseudomonas aeruginosa* ATCC 27853. A panel of 12 antimicrobial agents (HiMedia, Mumbai, India) was tested.

As CLSI does not provide disc diffusion criteria for *Acinetobacter*, polymyxin B minimum inhibitory concentrations (MICs) were determined by microbroth dilution and interpreted as per recommendations; intermediate results were categorized as resistant.

Multidrug-resistant (MDR) isolates were defined as non-susceptible to ≥1 agent in ≥3 classes; extensively drug-resistant (XDR) isolates as non-susceptible to ≥1 agent in all but ≤2 classes; and pan drug-resistant (PDR) isolates as non-susceptible to all tested agents [7].

Statistical analysis

Data were analyzed using Statistical Package for the Social Sciences (SPSS) Statistics version 21 (IBM Inc., Armonk, New York) [8]. Categorical variables were compared using the chi-squared or Fisher's exact test, and age using the t-test or Mann-Whitney U test.

Multiple comparisons were adjusted using the Benjamini-Hochberg false discovery rate (FDR). Penalized (Firth) logistic regression was applied for sparse data. A two-sided p-value < 0.05 was considered statistically significant.

## Results

During the six‑month study window, 181 *Acinetobacter* isolates were analyzed. Patient ages ranged from 0.02 to 87.0 years (mean 49.7; median 51; interquartile range 35-64). Males accounted for 122 (67.4%) and females for 59 (32.6%) isolates. The largest departmental contributions came from emergency medicine, 54 (29.8%), internal medicine, 41 (22.6%), and general surgery, 27 (14.9%). Most isolates originated from intensive care unit (ICU) settings, 143 (79.0%), with 35 (19.3%) from general wards and three (1.7%) from outpatient encounters, reflecting a substantial ICU burden.

Specimens were predominantly endotracheal aspirates, 100 (55.2%), followed by pus, 42 (23.2%), and blood, 25 (13.8%); the remaining 14 (7.7%) comprised sputum, urine, cerebrospinal fluid, and pleural fluid.

Subspecies distribution showed *A. baumannii*, 118 (65.2%),* A. lwoffii*, 39 (21.5%), and *A. haemolyticus*, 24 (13.3%), confirming* A. baumannii *as the predominant clinical species. This pattern aligns with the high proportion of respiratory specimens from mechanically ventilated patients, consistent with *A. baumannii *'s ecological advantage in airway devices and other biofilm‑rich interfaces.

Resistance rates were high across multiple classes. Among β‑lactams, cephalosporins, and aminoglycosides, resistance was 149 (82.3%) for piperacillin-tazobactam, 145 (80.1%) for ceftazidime, and 150 (82.9%) for gentamicin (Table 1). Carbapenem resistance remained substantial, with 145 (80.1%) for meropenem and 124 (68.5%) for imipenem (Table 2). In contrast, last‑line agents retained activity: polymyxin B showed 168 (92.8%) susceptibility with two (1.1%) resistance, while tigecycline susceptibility was 172 (95.0%).

**Table 1:** Antimicrobial susceptibility and resistance profile of Acinetobacter isolates (N=181)

Antimicrobial	Resistant, n (%)	Susceptible, n (%)
Piperacillin-tazobactam	149 (82.3)	—
Ceftazidime	145 (80.1)	—
Gentamicin	150 (82.9)	—
Meropenem	145 (80.1)	—
Imipenem	124 (68.5)	—
Polymyxin B	2 (1.1)	168 (92.8)
Tigecycline	—	172 (95.0)

**Table 2:** Comparison of carbapenem resistance between ICU and non-ICU isolates NS - not statistically significant; ICU - intensive care unit

Antibiotic	ICU resistant, n (%)	Non-ICU resistant, n (%)	p-value
Meropenem	116 (81.1)	29 (76.3)	0.667
Imipenem	95 (66.4)	29 (76.3)	NS

**Figure 1:**
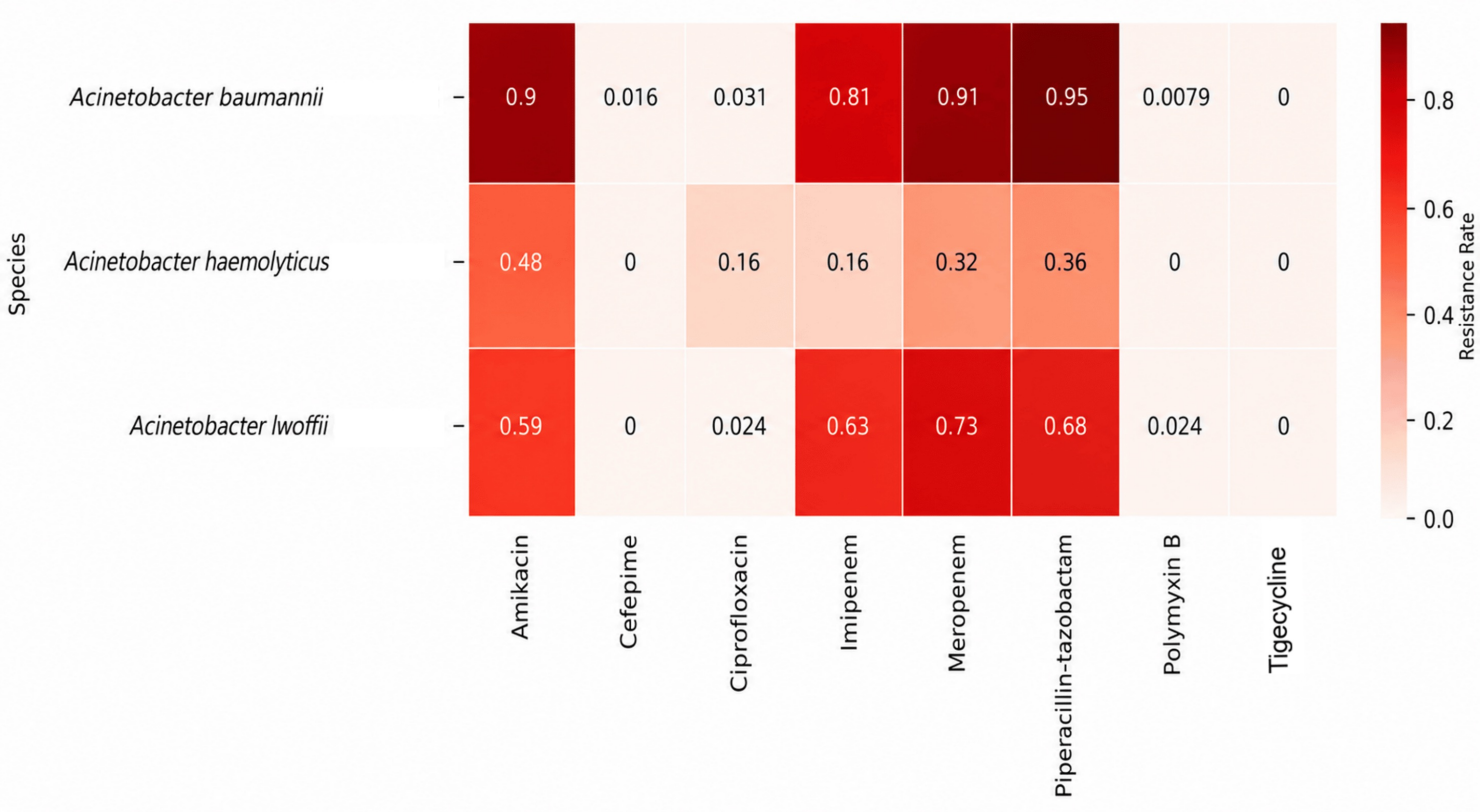
Heatmap of antimicrobial resistance Colour intensity corresponds to resistance magnitude across species and drug classes.

ICU versus non‑ICU comparisons showed no significant differences in carbapenem resistance (meropenem 81.1% vs 76.3%, p = 0.667; imipenem 66.4% vs 76.3%, not significant)(Table 2). Resistance categories differed significantly among species (χ², p ≈ 4.8 × 10⁻⁹). Among *A. baumannii*, multidrug resistance (MDR) was observed in 102 isolates (86.4%) and an extensively drug-resistant (XDR) phenotype in 16 isolates (13.6%). In *A. lwoffii*, MDR was identified in 32 isolates (82.1%) and XDR in one isolate (2.6%). For *A. haemolyticus*, MDR was present in 15 isolates (62.5%), with no XDR detected; nine isolates (37.5%) were non-MDR and demonstrated comparatively better susceptibility, particularly to cefepime (Tables 3-4, Figure 1).

**Table 3:** Distribution of resistance categories according to species MDR - multi-drug resistant; XDR - extensively drug-resistant Association between species and resistance category: χ² test, p = 4.8 × 10⁻⁹.

Species	MDR, n (%)	XDR, n (%)	Non-MDR, n (%)
*Acinetobacter baumannii *(n = 118)	102 (86.4)	16 (13.6)	0 (0.0)
*Acinetobacter lwoffii* (n = 39)	32 (82.1)	1 (2.6)	6 (15.4)
*Acinetobacter haemolyticus* (n = 24)	15 (62.5)	0 (0.0)	9 (37.5)

**Table 4:** Summary of key statistical findings R - resistance; S - sensitive; MDR - multi-drug resistant; XDR - extensively drug-resistant; ICU - intensive care unit *Clinical department that submitted the sample

Analysis parameter	Result / interpretation
Age vs MDR/XDR status	No significant difference (Mann-Whitney p = 0.185)
Gender vs MDR/XDR	No association (χ² p = 0.908)
Gender vs species	No association (p = 0.625)
ICU vs non-ICU (MDR/XDR)	Not significant (p = 0.816)
ICU vs non-ICU (Meropenem R)	Not significant (p = 0.667)
*Department vs MDR/XDR	Significant heterogeneity (p = 0.028)

Three points aid interpretation. First, the high resistance to legacy agents limits the usefulness of traditional β‑lactam and aminoglycoside regimens as empiric choices for suspected *Acinetobacter* infections in this setting. Second, although carbapenem resistance is widespread, the absence of an ICU versus non‑ICU difference suggests that resistance pressures extend beyond critical care, likely reflecting antimicrobial exposure patterns in upstream wards and emergency areas. Finally, the preserved activity of polymyxin B and tigecycline requires strict stewardship, including early cultures, expedited AST reporting, and tightly controlled indications to minimize toxicity and further resistance.

Demographic factors, including age, gender, ICU admission, and meropenem resistance, showed no significant association with MDR or XDR patterns (Table 4). The marked heterogeneity across departments likely reflects variations in case mix and device use. Emergency medicine, the largest source of isolates, frequently manages patients with prior healthcare exposure and empiric antibiotics, while general surgery more often encounters device‑related wound and drain infections. Although causality cannot be inferred, this variability reinforces the need for unit‑specific antibiograms and targeted stewardship feedback.

**Figure 2:**
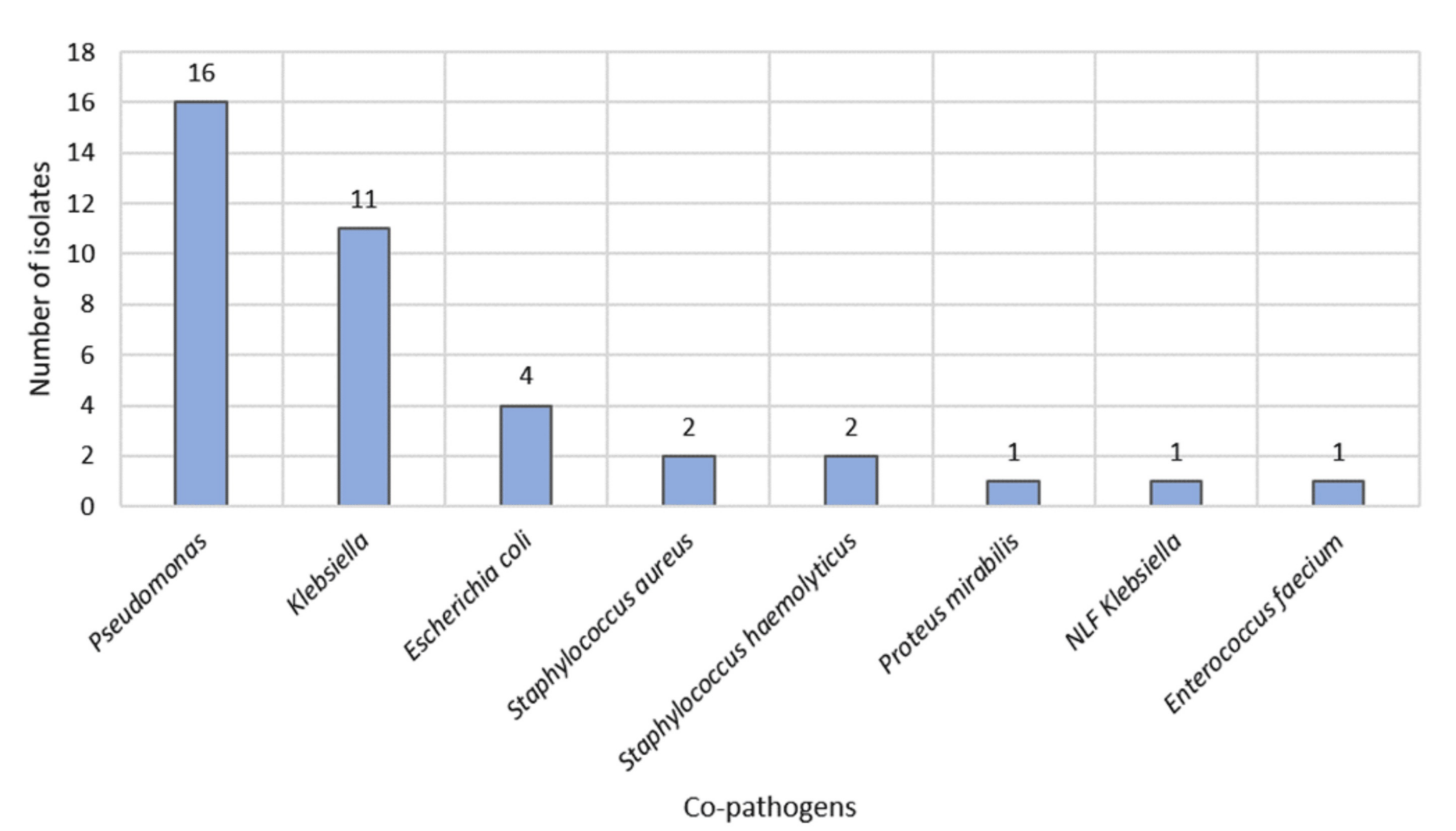
Co-pathogen bar chart NLF - non-lactose fermenting

Using a strict definition, 28 of 181 cultures (15.5%) were polymicrobial (Figure 2). The most common co‑pathogens were *Pseudomonas*, 11 (39.3%), *Klebsiella*, seven (25.0%), and *Escherichia coli*, three (10.7%), consistent with device‑associated and respiratory flora typical of critical care. Polymicrobial rates differed by species, with higher rates in *A. lwoffii*, 11 (28.2%), compared to *A. baumannii*, 13 (11.0%), yielding a significant species-polymicrobial association (χ² p = 0.036). Ward‑level variation showed a non-significant trend, and no associations were observed with sex (χ² p = 1.000) or age (Mann-Whitney p = 0.908). These findings suggest that polymicrobial isolation reflects species ecology rather than patient demographics.

Clinically, the frequent co‑isolation of non‑fermenters and *Enterobacterales* supports broad initial coverage in select severe presentations, with subsequent de‑escalation guided by organism‑specific susceptibilities.

## Discussion

This six-month prospective evaluation shows that *Acinetobacter* poses a considerable clinical burden in a tertiary care setting, with a clear predominance of ICU cases. *A. baumannii* emerged as the most frequent subspecies, a trend consistently reported in previous studies [8,9]. The antimicrobial resistance profile was high across several drug groups, particularly cephalosporins, aminoglycosides, and carbapenems, reflecting strong antimicrobial selection pressure and the well-established capacity of* A. baumannii* to persist in hospital environments, acquire diverse resistance genes, and spread through clonal transmission [10,11]. Within this setting, the continued in vitro effectiveness of polymyxin B and tigecycline offers important last resort treatment choices, although their clinical application requires strict antimicrobial stewardship and close monitoring for potential toxicity.

The lack of a measurable difference between ICU and non-ICU areas in carbapenem resistance may demonstrate a ceiling effect in which resistance has become equally widespread in both critical care and general ward settings during the study period. This pattern supports the need for stewardship strategies applied across the entire healthcare system rather than limiting interventions to ICUs. Such strategies include reassessing empiric treatment pathways, prioritizing culture collection before starting antimicrobials, and implementing rapid antimicrobial susceptibility testing methods to reduce time to de-escalation. The clear gradient in MDR and XDR across species, with the highest levels seen in *A. baumannii*, is consistent with its known epidemiologic tendencies for environmental persistence and clonal dissemination [12,13]. In contrast, *A. haemolyticus* showed no XDR and had a larger proportion of non-MDR isolates; when clinically appropriate, this difference can guide earlier de-escalation once species-level identification becomes available [14].

Our polymicrobial findings (15.5% overall; enriched for* Pseudomonas* and *Klebsiella*) are consistent with biofilm-associated infections and device-rich care settings [14,15]. Such contexts complicate the interpretation of causality and clinical significance, as *Acinetobacter* may act as a true pathogen, a colonizer, or a bystander within a polymicrobial community. Nevertheless, from a stewardship perspective, initial regimens must cover the most likely co-pathogens in unstable patients, with prompt refinement guided by culture results, source control, and pharmacodynamic targets. These principles are particularly important for respiratory sources, where distinguishing colonization from infection is challenging and where failure to provide adequate early coverage can lead to increased morbidity.

Our results indicate several key priorities. Maintaining subspecies-level reporting together with unit-specific antibiograms, combined with rapid workflows that connect early culture results to structured, time-bound reassessment of broad empiric therapy, can help reduce unnecessary carbapenem exposure [16]. Moreover, implementing standardized stewardship practices for last-line agents with an emphasis on safety, along with expanding the use of molecular epidemiology, can improve recognition of clonal transmission and evolving resistance patterns in high-risk units [17].

The study has several limitations. Being a single-center, ICU-weighted, laboratory-based investigation restricts the generalizability of the findings. Identification methods relied solely on phenotypic testing and VITEK, without molecular confirmation, limiting assessment of clonal relationships and resistance mechanisms [18]. Evaluation at the specimen level may have resulted in the over-representation of repeated isolates and did not account for patient-level clinical exposures. Additionally, the six-month duration precludes assessment of seasonal variation and long-term trends. Future studies should incorporate patient-level data, device-day denominators, molecular typing, and extended surveillance periods to better define epidemiology and resistance evolution.

## Conclusions

Across a six-month period in a tertiary care setting,* Acinetobacter* spp. posed a significant clinical burden, predominantly in ICU patients, with *A. baumannii* as the leading subspecies. High antimicrobial resistance limited reliable treatment options to polymyxin B and tigecycline. Species-level differences were evident, with *A. baumannii* showing the highest MDR and XDR rates, supporting the need for species-specific empiric therapy and timely de-escalation.

Polymicrobial infections (15.5%), most commonly involving *Pseudomonas* spp. and *Klebsiella* spp. alongside *Acinetobacter* spp., highlight the complexity of device-associated infections, particularly in the context of multidrug-resistant organisms. These findings underscore the critical importance of robust infection control practices and continuous surveillance, including subspecies-level identification. The comparatively lower resistance rates observed in *A. lwoffii* and *A. haemolyticus* further emphasize the variability in antimicrobial susceptibility across *Acinetobacter *subspecies.

Overall, these findings reinforce the need for targeted antimicrobial stewardship, optimized empiric therapy, and strengthened infection prevention strategies to limit further resistance.
